# High level expression of bikunin in *Pichia pastoris *by fusion of human serum albumin

**DOI:** 10.1186/2191-0855-2-14

**Published:** 2012-02-29

**Authors:** Xing-Hua Gou, Yu-Ying Liu, Qi-Lei Chen, Jian-Jun Tang, Da-Yu Liu, Liang Zou, Xiao-Yong Wu, Wei Wang

**Affiliations:** 1 Faculty of Bioindustry, Chengdu University, Waidong Shilingzhen, Chengdu, 610106, China; 2 Genekey Biotech. (Chengdu) Co., Ltd. Chengdu, 610041, China; 3 Meat Proceeding Key Lab. of Sichuan Province, 610106, China

**Keywords:** Human bikunin, Fusion expression, Human serum albumin, *Pichia pastoris*

## Abstract

Bikunin is a proteoglycan exhibiting broad-spectrum inhibitory activity against serine proteases and could potentially suppress tumor cell invasion and metastasis. Here, we have successfully expressed recombinant human bikunin (rh-bikunin) in *Pichia pastoris *and also established the purification procedure. Different fusion genes of h-UTI and domain I, domain I and domain II, domain I, domain II and domain III of human serum albumin (HSA) were inserted into expression vector pPICZαA. After expressed in shake flask, rh-bikunin was produced in an 30-L fermenter and purified by affinity chromatography and cation exchange chromatography. The final expression levels were 200 mg/L and we got totally 1.08 g (3650 IU/mg) of active purified rh-bikunin (purity is 98%) from 20 L of fermentation broth. The rh-bikunin consists of unique form with molecular masses of 25 kDa, and has the same N-terminals sequence as human native bikunin. This study provided a new method for high level expression of active rh-bikunin by using HSA as fusion parter.

## Introduction

Bikunin, also being called urinary trypsin inhibitor (UTI), contains two antiproteolytic Kunitz domains. The protein is a proteoglycan ([Xu, Carr et al. 1998]), which has a molecular mass of about 25 kDa including a 6-7 kDa chondroitin sulfate chain ([Pugia, Valdes Jr et al. 2007]; [Chi, Wolff et al. 2008]). Bikunin is synthesized in the liver together with another plasma protein, α_1_-microglobulin (α_1_-m), forming a precursor (α_1_-m/bikunin precursor, AMBP). As a kind of serine proteinase inhibitor, bikunin exhibits broad inhibitory activity against many proteases, such as trypsinase, chymotrypsin, leukocyte elastase, and fibrinolytic enzyme. Moreover, human bikunin hasn't antigenicity to human and has the characteristic of use safety, so it has been widely used as a drug for patients with acute pancreatitis, acute attack of chronic pancreatitis, acute circulation exhaustion, tumor and shock ([H Inaba 1986]; [Okuhama, Shiraishi et al. 1999]; [Kobayashi, Suzuki et al. 2003]; [Yano, Anraku et al. 2003]; [Molor-Erdene, Okajima et al. 2005]; [Qing xia 2005]; [Zhang, Liu et al. 2011]). The bikunin has many advantages such as evident effect in clinic, low side effect and low production cost. However, due to the low content in urinary, difficult collection of human urinary and high cost of purification, the bikunin is limited to apply widely. To overcome these problems, a promising alternative technique is to obtain recombinant human bikunin by gene recombination.

The bikunin have been successfully cloned and expressed in *E. coli *and *Pichia pastoris *([Fritz 1995]; [Brinkmann, Weilke et al. 1997]; [Jian-qiu, Feng-qin et al. 2008]). However, the yield of recombinant human UTI (rh-UTI) in *E. coli *or *P. pastoris *is too low and the uniform of protein doesn't to be ensured. There hasn't been report about large scale production and animal model examination so far. Therefore, the clinic value of rh-UTI is difficult to be determined all the same.

Previous study showed that the use of human serum albumin (HAS) as N-terminal fusions can be an effective technique to express difficult proteins in mammalian cells ([Carter, Zhang et al. 2010]; [Zhang, Carter et al. 2010]). So in this study, fusion genes of h-UTI and domain I, domain I and domain II, domain I, domain II and domain III of human serum albumin were inserted into expression vector pPICZαA, respectively. Finally, all plasmids were linearized for transformation into *P. pastoris *strain GS115. The h-UTI was highly expressed in *P. pastoris*, which successfully solved the problems of the uniform and low yield of h-UTI expressed in *P. pastoris*.

## Materials and methods

### Strains, vectors and other reagents

The *P. pastoris *GS115, pPICZαA vector, and Zeocin antibiotic were obtained from Invitrogen (CA, USA). *P. pastoris *were grown in YPD medium containing 10 g/L yeast extract, 20 g/L peptone, and 20 g/L D-glucose. To prepare YPD plates, 2% agar (w/v) was added to the YPD medium. YPD-Zeocin plates (1% yeast extract, 2% peptone, 2% dextrose, 2% agar, and 0.1-0.2 mg/mL Zeocin) were used for selecting multicopy transformants. The *P. pastoris *cells were cultured in BMGY medium (1% yeast extract, 2% peptone, 1% glycerol, 400 μg/L biotin, and 0.1 M potassium phosphate, pH 6.0) for growth and in BMMY medium (1% yeast extract, 2% peptone, 400 μg/L biotin, 1% methanol, and 0.1 M potassium phosphate, pH 6.0) for induction. All primers were synthesized by Sangon Biotechnology Corp. (Shanghai, China). All restriction enzymes, DNA marker, synthesized genes (human BSA-UTI fusions) and protein marker were purchased from Takara (Dalian, China). The standard human UTI, trypsin purchased from Sigma-Aldrich (St. Louis, USA).

### Construction of expression vector pPICZα-HSA-UTI

**Figure 1:**
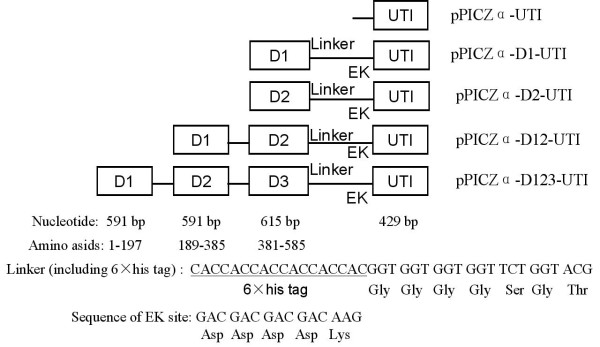
**Maps of fusion genes**. Plasmid pPICZαA was used as a parent vector for constructing these fusions. D1, D2 and D3 represent different domain of human serum albumin (HSA) gene. Several Gly residues designed as linker. EK represent the site of enterokinase. UTI represent the gene of urinary trypsin inhibitor.

Construction of rh-bikunin expression vector was based on pPICZαA vector. The synthesized human serum albumin (HSA) gene containing different domains were added 6 × His tag, several Gly linkers and the recognition site of Enterokinase (EK) at the C-terminal (as shown in Figure 1). The synthesized HSA gene and UTI gene were ligated to pMD18-T vector. The recombinant plasmids pMD-HSA-UTI were digested with *Xho *I and *Not *I and then inserted into the same site of pPICZαA. The recombinant expression plasmids were designated as pPICZα-UTI, pPICZα-D1-UTI, pPICZα-D2-UTI, pPICZα-D12-UTI and pPICZα-D123-UTI, respectively.

### Transformation of *P. pastoris *and selection of high-level expression

The five *Sac *I restriction endonuclease linearized recombinant expression vectors were introduced into *P. pastoris *GS115 by electroporation using a Micropulser (Bio-Rad, USA) according to the manual, respectively. Transformed cells were selected by growth on yeast extract peptone dextrose (YPD) agar plates containing Zeocin (0.5 mg/mL). After the multicopy transformants appeared, single clone was cultured in 5 mL BMGY medium at 28°C with shaking at 250 rpm for 24 h. The cells were then centrifuged and resuspended in 5 mL BMMY medium to induce expression for 4 days. The culture medium (0.5 mL) was sampled per day and centrifuged at 4°C, 10,000 rpm for 5 min. Cell pellet and supernatant were separated. The supernatant was tested for UTI activity and cell pellet was used for genomic DNA analysis. pPICZα A blank plasmids were also transformed as a negative control.

### Optimization of fermention conditions by shake-flask mode

In order to determine the effect of pH on the expression level of rh-UTI, the BMGY media was performed at different pH values (pH 4.0, 5.0, 5.5, 6.0, 6.5, 7.0). In order to determine the effect of feeding mode on the expression level of rh-UTI, inorganic salt, peptone, yeast extracts and glycerol was used as the carbon sources in BMGY media by continuous fed-batch mode, respectively.

### High-density fermentation

A stock culture of *P. pastoris *was grown to an A_600 _of 3-6 in a 5-L shake flask containing 2 L YPD. The shake flask culture was used to inoculate an 30-L fermenter (Bioengineering, AG) containing 20 L of fermentation basal salts medium FM22 supplemented with PTM1 trace salts (1.1 mL of stock solution/L) and biotin (0.4 mL of the stock solution/L). The dissolved oxygen level (DO) was set at 30% and the stirring rate was 700 rpm. The pH of the medium was maintained 6.0-6.5 by automatic addition of 5 N NH_4_OH and 1 M phosphoric acid and 5% antifoam as required. Temperature was maintained at 30°C. The initial cultivation terminated when all glycerol was consumed (about 14 h) at batch phase. Continuous 2% peptone and 1% yeast extract feeding were carried out for about 6 h in the subsequent fed-batch phase. To induce rh-UBI expression, 100% methanol was fed at 3 mL/h/L for 50 h. Sampling of the culture medium at the end of each phase was performed for assay of rh-bikunin activity.

### Purification of rh-UBI and assay of trypsin inhibitory activity

The supernatant was collected by centrifugation, 5 M NaCl and phosphate buffer was added to a final concentration of 1 M and 50 mM, respectively. Finally, the pH value was also adjiusted to 7.4 with NaOH. Then the treated supernatant was was clarified with a 0.45 μm cellulose membrane. The supernatant was purified with chelating sepharose affinity chromatography and Q sepharose F. F. anion exchange chromatography in turn. Then after digested by enterokinase, the sample was isolated and purified by chelating sepharose affinity chromatography and SP sepharose F.F cation exchange chromatography. The purity of each step protein sample was checked by SDS-PAGE. The final rh-bikunin protein was stored at -20°C for further assay of trypsin inhibitory activity accroding to previous study ([Page, Quillien et al. 2000]; [Wang, Yan et al. 2008]).

### Carbohydrate digestion and amino-terminal sequence

The amino-terminal sequence of rh-bikunin was determined automated Edman degradation method (Shanghai Sangon Biological Engineering Technology & Services CO., Ltd). N-linked glycosylation was assayeded by digestion of the protein with N-glycosidase F. Protein samples (20 μg) were boiled for 5 min in 1% (w/v) SDS and 50 mM DTT. Samples were then diluted to 0.1% SDS, 5 mM DTT, 20 mM sodium phosphate (pH 7.4), 25 mM EDTA, and 2% Triton X-100. N-glycosidase F (0.8 U) was added and samples were incubated at 37°C overnight. Deglycosylation of the protein was assessed by a shift in electrophoretic mobility on SDS-PAGE analysis.

## Results

### Construction of recombinant plasmids and identification of transformed colonies

Five plasimds containing of different domain of HSA gene were constructed (Figure 1). DNA sequence analysis of the five recombinant expression vectors pPICZα-HSA-UTI demonstrated that cDNA encoding human bikunin and human serum albumin (HSA) gene were correctly inserted into pPICZαA vector. The recombinant expression vectors were then linearized with *Sac *I and introduced into *P. pastoris *strain GS115. Agarose gel electrophoresis of the PCR products showed that cDNA encoding bikunin was indeed integrated into the the *P. pastoris *genome. However, there were no visible bands from the control sample which were transformed with pPICZαA blank plasmid.

### Shake-flask expression of rh-bikunin

Three clonies containing of pPICZα-D12-UTI showed the highest expression level in shake-flask expression than other recombinant plasmids (Data not shown). The effects of pH value and carbon sources on the expression level of rh-UTI in *P. pastoris*/pPICZα-D12-UTI were also investigated by shake-flask fermentation. The pH value of BMGY media was adjusted to the desired value. The results showed that the expression levels of rh-bikunin were different when pH of medium varied from 4.0-7.0. When the pH value was 6.0-6.5, the expression level of rh-UBI was higher than those of other pH values. In order to determine the effect of feeding mode on the expression level of rh-UBI, inorganic salt, peptone, yeast extracts and glycerol was used as the carbon sources in BMGY media by continuous fed-batch mode, respectively. The results displayed that when the inorganic salt, peptone and yeast extracts were used as the carbon sources in BMGY media, the expression level of rh-UBI was higher than that of glycerol. Therefore, the optimal pH (6.0-6.5) and carbon sources (inorganic salt, peptone or yeast extracts) were selected as shake-flask condition and further fermentation (data not shown).

### High-density fermentation and purification of the recombinant protein

**Figure 2:**
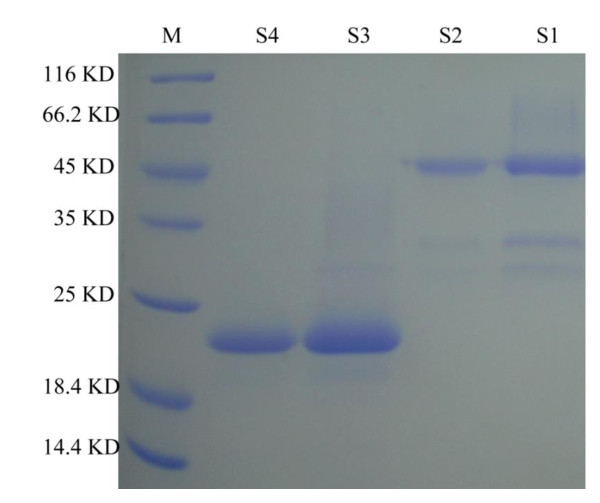
**SDS-PAGE of the recombinant rh-UTI in different step of purification**. S1, Chelating sepharose affinity chromatography; S2, Q sepharose F.F. anion exchange chromatography; S3, Chelating sepharose affinity chromatography after digested by enterokinase; SP sepharose F.F cation exchange chromatography.

Under optimal conditions (pH 6.0, 30°C, and DO set at about 30%), the highest optical density and cell wet weight were obtained after 50 h of methanol induction. The expression level was also reached to 200 mg/L. The rh-bikunin solution was purified with chelating sepharose affinity chromatography and Q sepharose F.F. anion exchange chromatography in turn. Then after digested by enterokinase, the sample was isolated and purified by chelating sepharose affinity chromatography and SP sepharose F.F cation exchange chromatography. The protein recovery of the rh-bikunin at the different purification steps were summarized in Table 1. Following these processes, we could get totally 1.08 g of purified rh-bikunin (purity is 98%) from 20 L culture medium as revealed by SDS-PAGE (Figure 2 and Table 1). Rh-bikunin was secreted in the medium after induction and a final concentration of approximately 3650 IU/mg was obtained, which was 1.46 times higher than that of native human urinary bikunin (2500 IU/mg).

**Table 1:** Summary of purification process of rh-UTI

*Purification step*	*Volume* *(mL)*	*Protein concentration* *(mg/L)*	*rh-UTI* *(mg)*	*Protein recovery (%)*
Supernatant	20,000	200	4,000	
Ultrafiltrated	3,000	1.20	3,600	90.0
Chelating sepharose	600	4.75	2,850	71.25
Q sepharose F.F.	350	6.68	2,338	58.45
Chelating sepharose after digested by Enterokinase	1,500	0.90	1,350	33.75
SP sepharose F.F	220	4.91	1,080	27.0

### Characterization of purified rh-UTI

**Figure 3:**
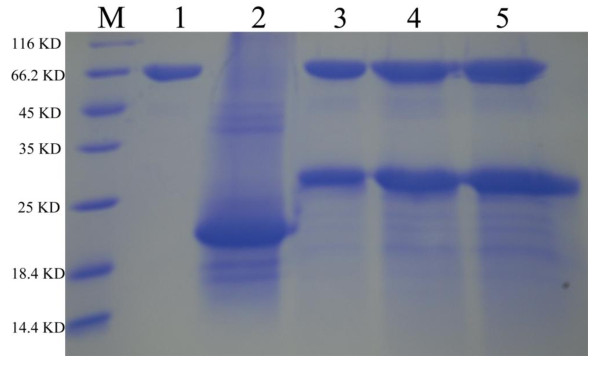
**Deglycosylation of rh-UTI**. Purified recombinant human bikuninwas treated with N-glycosidase F and examined by SDS-PAGE. Lanes 1, negative control; Lanes 2, untreated rh-UTI; Lanes 3, 4 and 5, after treatment with N-glycosidase F. For rh-UTI, the mass, calculated from the migration distance in SDS-PAGE, increased by approximately 5 kDa after treatment with N-glycosidase F (Lanes 3, 4 and 5).

**Figure 4:**
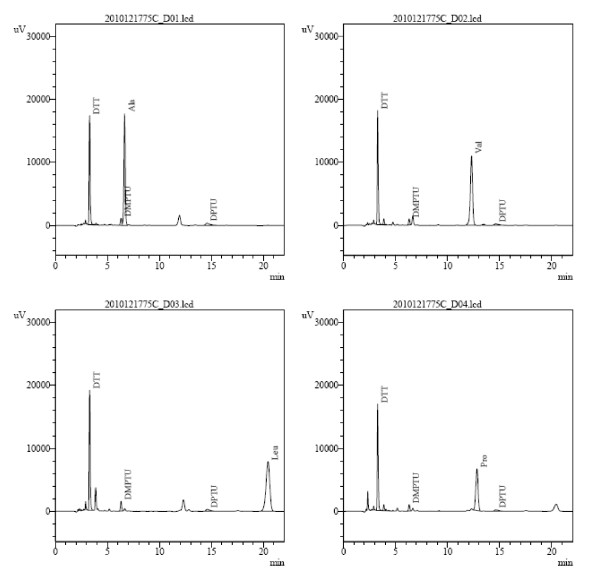
**Amino-terminal sequence of rh-UTI**. N-terminus of rh-bikunin was Ala-Val-Leu-Pro.

According to SDS-PAGE analysis (Figure 2, Lanes S3 and S4), rh-UTI migrated as a single band. The molecular masses based on protein migration rates in SDS gel were 23~24 kDa. However, double band was observed in other previous study ([Falkenberg, Wester et al. 2001]; [Wang, Yan et al. 2008]). The mass of rh-UTI increased by approximately 5 kDa after treatment with N-glycosidase F (Figure 3, Lanes 3, 4 and 5). On the other hand, the N-terminal amino acid sequence of rh-UTI was also identical to the N-terminus of native human bikunin (Figure 4, Ala-Val-Leu-Pro), which demonstrating correct processing ([Kakizaki, Takahashi et al. 2007]).

## Discussion

The expression system of yeast *P. pastoris *has several advantages, including the use of the alcohol oxidase I (AOX1) gene promoter, the ability of the cells to be cultivated at high density, a simplified purification procedure for secreted heterologous proteins, and modifications of foreign proteins ([Wang, Yan et al. 2008]). So, the expression system of yeast *P. pastoris *was used for bikunin expression. On the other hand, several parameters such as clone selection, codon optimization, fusion partners and culture conditions play a important role in obtaining high yields of protein. So, in order to optimize the level of protein production experiment, we evaluated the role of codon optimization, fusion partners and culture conditions in obtaining high yields of bikunin.

Firstly, codon bias is an intrinsic problem in heterologous protein production and needs to be taken into account in the experimental design. So, the synthesized human serum albumin (HSA) gene was used in this study. Secondly, previous study showed that the use of human serum albumin (HAS) as N-terminal fusions can be an effective technique to express difficult proteins in mammalian cells ([Carter, Zhang et al. 2010]; [Zhang, Carter et al. 2010]). So in this study, fusion genes of h-UTI and domain I, domain I and domain II, domain I, domain II and domain III of human serum albumin were inserted into expression vector pPICZαA, respectively (As shown in Figure 1). The results showed that three clonies containing of pPICZα-D12-UTI (containing domain I and domain II) showed the highest expression level in shake-flask expression than other recombinant plasmids. Thirdly, the effects of pH value and carbon sources on the expression level of rh-UTI in *P. pastoris*/pPICZα-D12-UTI were also investigated by shake-flask fermentation. Rh-bikunin was secreted in the medium after induction and the final expression levels were 200 mg/L. As a result, we got totally 1.08 g (3650 IU/mg) of active purified rh-bikunin (purity is 98%) from 20 L of fermentation broth, which was 1.46 times higher than that of native human urinary bikunin (2500 IU/mg) ([Wang, Yan et al. 2008]). By combined these methods, we sucessfully expressed active rh-bikunin at the high level.

## Competing interests

The authors declare that they have no competing interests.
